# MDM2-amplified esophageal adenocarcinomas exhibit an activated metabolic and immunosuppressive phenotype with multiple potential therapeutic targets

**DOI:** 10.1186/s12885-025-15367-3

**Published:** 2025-11-26

**Authors:** Karl Knipper, Christiane J. Bruns, Felix C. Popp, Thomas Schmidt, Bastian Grothey, Alexander Quaas, Su Ir Lyu

**Affiliations:** 1 Faculty of Medicine and University Hospital of Cologne, Department of General, Visceral, Thoracic and Transplantation Surgery, University of Cologne, Cologne, Germany; 2 Faculty of Medicine and University Hospital of Cologne, Institute of Pathology, University of Cologne, Cologne, Germany

**Keywords:** Esophageal adenocarcinoma, MDM2, Amplification, Prognosis, Proteomics, Personalized medicine

## Abstract

**Background:**

A complete response following neoadjuvant therapy occurs only in a fraction of patients with esophageal adenocarcinoma (EAC). Further targeted treatment options are needed to improve treatment response and patient survival. Mouse double minute 2 homolog (MDM2) is a known oncogene. It is currently under investigation as a potential therapeutic target. However, initial findings have not been groundbreaking. Therefore, this study aimed to identify further potential therapeutic targets in the subtype of MDM2-amplified EAC.

**Methods:**

We screened 656 patients with esophageal adenocarcinoma for their MDM2-amplification status using Fluorescence in situ Hybridization. 57 tumors (8.7%) were MDM2-amplified. The proteome of 35 MDM2-amplified and 37 non-amplified tumors was analyzed using mass spectrometry.

**Results:**

The comparison of MDM2-amplified and non-amplified tumors revealed various differently expressed proteins and pathways. Hornerin and Bystin were significantly downregulated. Whereas Choline transporter-like protein 2 and Contactin 1 were upregulated in MDM2-amplified EAC. Enrichment analyses revealed that MDM2-amplified EAC showed a more pronounced immunosuppressive phenotype. Furthermore, distinct metabolic pathways like propanoate and tryptophan metabolism were upregulated.

**Conclusions:**

Summarizing, we described numerous potential therapeutic targets, suggesting patients with MDM2-amplified tumors could potentially benefit from, exemplary, Mitogen-activated protein kinase kinase inhibitors or tryptophan metabolism inhibitors in new combinational treatment regimens. Future mechanistic studies are needed to validate these findings.

**Supplementary Information:**

The online version contains supplementary material available at 10.1186/s12885-025-15367-3.

## Background

Multimodal therapy is the standard of care for patients with locally advanced esophageal adenocarcinoma [1]. In 2024, the ESOPEC trial showed that patients who underwent perioperative chemotherapy according to the FLOT protocol (Fluorouracil, Leucovorin, Oxaliplatin, Docetaxel) had a significantly better survival rate than those who received neoadjuvant chemoradiotherapy. While the 3-year overall survival could be increased to 57.4%, a complete therapy response could be observed in only 19.3% of the patients treated with FLOT [2]. It is evident that perioperative treatment options must be improved to further increase patients’ survival.

Mouse double minute 2 homolog (MDM2) is a p53-specific E3 ubiquitin ligase. MDM2 monoubiquitinates p53. This step is followed by the degradation of p53 by proteasomes [3]. Therefore, MDM2 is a natural counterpart of p53. Under physiological conditions, MDM2 is upregulated in a p53-dependent manner. MDM2 inhibits p53 and increases its degradation, so p53 cannot initiate cell-cycle arrest or apoptosis [4]. Therefore, *MDM2* is defined as an oncogene [4]. Structural and functional studies show that blocking the MDM2-p53 interface stabilizes p53 and restores its transcriptional program, providing a clear druggable axis. Multiple small-molecule inhibitors (e.g., nutlins, idasanutlin/siremadlin, APG-115) validate this concept preclinically and have entered clinical testing across solid tumors [5].

The serum concentration of MDM2 can be upregulated in various ways in tumors. This could occur at the transcriptional level, for instance, through gene amplification, or at the post-transcriptional level, such as phosphorylation and subsequent stabilization of MDM2 by various kinases [6]. MDM2 amplification occurs in many tumor entities. The frequency of MDM2 amplification varies between nearly 100% in well-/de-differentiated liposarcomas and 0.23% in adenocarcinomas of the appendix [7]. MDM2 amplification also occurs in esophageal adenocarcinoma in approximately 6% of all patients [7, 8]. While TP53 mutation is common in EAC, a distinct TP53-wild-type subset exhibits copy-number gain of MDM2. Importantly, TP53-wild-type EACs are enriched for MDM2 amplification, nominating them for p53-reactivation strategies with MDM2 inhibitors [9, 10]. Functionally, MDM2 overactivity phenocopies TP53 loss and is associated with poor response to cytotoxic therapy in gastro-esophageal disease [11]. Preclinical esophageal models show that pharmacologic MDM2 antagonism restores p53 function and sensitizes to chemotherapy [11].

MDM2 overexpression not only promotes cancer progression by inhibiting p53 but also acts as a pro-angiogenic stimulus through the upregulation of matrix metallopeptidase 9 and tumor necrosis factor-alpha [12]. Additionally, MDM2 activates the protein kinase B (Akt) pathway through Akt phosphorylation, resulting in improved cancer cell survival and increased epithelial-mesenchymal transition [13, 14]. Despite promising results of the treatment with immune checkpoint inhibitors, only a fraction of patients benefits from these new therapy agents in esophageal cancer [15]. MDM2 amplification is assumed to be one of the possible mechanisms of therapy resistance [16]. By accelerating p53 turnover, MDM2 blunts cell-intrinsic tumor suppressor pathways and attenuates p53-driven immunogenic programs, fostering an immune-cold phenotype [9]. MDM2 can support the immune evasion of the tumor by altering the immune microenvironment in a p53-dependent and -independent manner [17]. Exemplary, previous research could reveal a negative association between p53 expression and the expression of Programmed death-ligand 1 (PD-L1) [18]. Indicating, an MDM2-dependent downregulation of p53 would lead to an increased expression of PD-L1. MDM2 expression was reported to lead to a higher resistance to T cell-mediated tumor killing. Targeting Ovarian clear cell carcinoma cell lines with an MDM2 inhibitor showed the ability to overcome this resistance. It was shown that the protumorigenic Interleukin 6 was downregulated in MDM2-knockdown cells [19]. Interleukin 6 inhibits dendritic cells, helper T cells, and cytotoxic T cells via reduction of Interferon γ [20]. MDM2 amplification is associated with hyperprogression under immunotherapy. In a patient cohort with stage IV cancer patients, all six patients (bladder cancer, breast cancer, endometrial stromal sarcoma, lung adenocarcinoma, squamous cell carcinoma of the hypopharynx) with MDM2 amplification showed a time-to-treatment failure of under two months. Alarming, the tumor burden of these patients increased by up to 258% under immunotherapy [21]. Moreover, MDM2 amplification status could also be linked with poor response to other antiproliferative treatment agents. Patients with esophageal adenocarcinoma with MDM2 amplification showed a poor treatment response followed by neoadjuvant therapy in a retrospective trial, resulting in a significantly worse progression-free survival of these patients [22]. Additionally, MDM2´s potential as a therapeutic target is being evaluated in various tumor entities [5]. Preclinically, administration of APG-115, a small-molecule MDM2 inhibitor, enhanced the radiosensitivity of p53-wild-type gastric cancer cells in vitro. Moreover, in mouse models, combined treatment with APG-115 and radiation resulted in greater tumor growth reduction compared with either treatment alone [23]. In colon carcinoma models, treatment with HDM201, another MDM2 inhibitor, increased T-cell-mediated tumor cell killing in vivo. Additionally, the combination of HDM201 and a programmed death-1 (PD-1) checkpoint inhibitor further enhanced tumor regression in vivo. Interestingly, mice that responded to this therapy developed antigen-specific memory T cells, which led to rejection of subsequent tumor challenges [24]. Based on promising preclinical data, MDM2 inhibitors are also under investigation in clinical trials. For example, the administration of ALRN-6924, a dual inhibitor of MDM2 and MDMX, in a phase I study involving patients with solid tumors or lymphomas resulted in a response rate of 59%, with two patients demonstrating a complete response [25]. In another phase I trial of the MDM2 inhibitor alrizomadlin, conducted in patients predominantly diagnosed with advanced liposarcoma who had progressed on standard therapy, a partial response was achieved in 2 patients, while 10 patients experienced stable disease [26]. Milademetam, a selective small-molecule inhibitor of the MDM2-p53 interaction, was evaluated in a multicenter, single-arm phase II trial. The study enrolled patients with MDM2-amplified solid tumors, including, for example, sarcoma, breast cancer, and cholangiocarcinoma. An objective response was observed in 19.4% of patients, and the treatment demonstrated a manageable safety profile [27]. While additional clinical studies are currently recruiting, emerging evidence suggests that combined tumor therapy regimens result in better response rates and help overcome therapy resistance [28]. Together, these data support MDM2 as a tractable target in a biologically defined EAC subset. Therefore, we aimed to characterize the proteome of MDM2-amplified esophageal adenocarcinomas in comparison to non-amplified tumors, identifying altered pathways as potential additional therapeutic targets.

## Materials and methods

### Patients and tumor samples

In this study, 656 patients were included. This patient cohort was used in previous publications [29–31]. Inclusion criteria were the diagnosis of an adenocarcinoma of the esophagus or the esophagogastric junction, resection at the University Hospital of Cologne with curative intent, and written informed consent to participate in our tissue bank. Exclusion criteria included histologies other than adenocarcinomas, an overall survival of less than 3 months, or poor tissue quality, which was not suitable for further analyses. Patients underwent thoracoabdominal esophagectomy, including lymphadenectomy, or transhiatal extended gastrectomy at the University Hospital Cologne from 2013 to 2019. Overall survival was defined as the time from surgery to death or loss of follow-up. Patient data were prospectively collected and retrospectively analyzed. Written informed consent to participate in our tissue bank was obtained from every individual. This study complied with the Declaration of Helsinki and was approved by the local ethics committee (protocol code: 20–1393).

Specimens were assessed following the 7th edition of the Union for International Cancer Control. Tumor-bearing specimens were fixed with formalin and embedded in paraffin. Using a semi-automatic precision instrument, 1.2 mm tumor cylinders were punched out of tumor areas and transferred onto tumor microarrays.

### Fluorescence-in-situ-hybridization (FISH)

FISH was performed as described previously [32]. In short, 4 μm sections of the paraffin-embedded tissue microarray were used. These sections were hybridized overnight with the Zyto-Light SPEC MDM2/CEN12 Dual Core Probe (ZytoVision, Bremerhaven, Germany). The fluorescent stainings were assessed with an x100 objective on a DM5500 fluorescent microscope with appropriate filter settings (Leica Biosystems, Wetzlar, Germany). Twenty tumor nuclei of three different areas were examined per tumor sample. Green (MDM2) and orange (centromere (CEN) signals were counted. MDM2 amplification was defined as ≥ 50% of the cells containing ≥ 5 MDM2 signals, ≥ 10% of the cells containing ≥ 15 gene copies, MDM2/CEN ratio ≥ 2.0, or an average MDM2 gene copy number per cell ≥ 6.0 [33]. Internal controls were conducted via the screening of lymphocytes or fibroblasts in the tumor microenvironment of the sample. Exemplary images of MDM2-amplified and non-amplified tumors can be found in Supplementary Fig. 1.

### Proteome analysis with mass spectrometry

The proteome of 35 MDM2-amplified and 37 MDM2-non-amplified tissue samples were analyzed with mass spectrometry. Mass spectrometry was performed as described before [34]. A detailed protocol is supplied in the Supplementary methods. Reproducibility was ensured by the analysis of pool samples every 16 samples. The proteomic data from the non-amplified and non-tumor tissue samples were used in previous publications [29]. The raw liquid chromatography-mass spectrometry data have been deposited to the ProteomeXchange Consortium via the PRIDE [35] partner repository with the dataset identifier PXD058762.

### Statistical analyses

The data were processed with MaxQuant (version 2.6.7.0, Max Planck Institute of Biochemistry, Martinsried, Germany). The following analyses were conducted as published before using Perseus (version 2.1.3.0, Max Planck Institute of Biochemistry, Martinsried, Germany) [36]. Differential expression was analyzed using ANOVA and Welch´s T-test. Proteins were annotated into clusters using the Kyoto Encyclopedia of Genes and Genomes (KEGG) pathway name, Gene Ontology Biological Process (GOBP) name, and Reactome name encyclopedia (downloaded on the 16th December 2024). The enrichment of distinct pathways was analyzed using the 1D enrichment analysis (full enrichment results are available in Supplementary Tables 3, 5, 7, 9) [37]. Multiple testing correction was done with the Benjamini-Hochberg FDR using a threshold value of 0.02. *P*-values < 0.05 and p-adjusted-values < 0.2 were defined as statistically significant. Qualitative clinicopathological variables were compared with the chi-square test using IBM SPSS Statistics (Version 29.0.1.1, IBM, Armonk, USA). MaxQuant and SPSS were used for graphical visualization.

## Results

### Overall survival showed no significant differences between patients with and without MDM2 amplification

In this study, 656 tumor samples were screened for MDM2 amplification. Therefore, FISH stainings were conducted on a tissue microarray. Here, 57 tumor samples (8.7%) showed an MDM2 amplification based on the criteria mentioned above. Out of this patient cohort, 35 MDM2-amplified tumor samples were randomly chosen to further analyze the proteome (Supplementary Table 1). As a reference group, 37 MDM2-non-amplified tumor samples were selected. To avoid any interference from different treatment regimens, the reference group was balanced in terms of the rate of neoadjuvant, perioperative therapy, or primary surgery, as neoadjuvant therapy is part of the standard of care and should also be represented in this study. In detail, 22.2% (*n* = 16) of all included patients were primarily resected, and 77.8% (*n* = 56) were neoadjuvantly treated before resection. In the MDM2-amplified cohort, seven patients (20.0%) were primarily resected, 28 patients (80.0%) were neoadjuvantly treated compared to 9 primarily resected patients (24.3%), and 28 neoadjuvantly treated patients (75.7%) in the non-amplified cohort. Furthermore, 20 non-tumoral tissue samples were included for internal quality control. The patient characteristics of the total cohort and the patients that were included in the proteome analyses are depicted in Tables 1 and 2.

**Table 1:** Patient characteristics of the total study cohort and patients with MDM2-non-amplified or MDM2-amplified tumors. (y)pN: pathological lymph node status (after neoadjuvant therapy), (y)pT: pathological tumor status (after neoadjuvant therapy), CROSS: chemoradiotherapy for oesophageal cancer followed by surgery Study, FLOT: fluorouracil, leucovorin, oxaliplatin, docetaxel, L: lymph vessel infiltration, Pn: perineural infiltration, V: blood vessel infiltration

Characteristic	Total	MDM2		
		non-amplified	amplified	
	*n* (%)	*n* (%)	*n* (%)	*p*-value
No. of patients	656 (100)	599 (100)	57 (100)	
Sex				0.057
Male	580 (88.4)	534 (89.1)	46 (80.7)	
Female	76 (11.6)	65 (10.9)	11 (19.3)	
Age				0.667
< 65	362 (55.2)	329 (54.9)	33 (57.9)	
≥ 65	294 (44.8)	270 (45.1)	24 (42.1)	
Median overall survival (months)	22.8	23.0	21.7	
(minimum-maximum)	(1.0–233.6.0.6)	(1.0–233.6.0.6)	(1.3–185.4.3.4)	
Neoadjuvant/perioperative therapy				0.054
No	213 (32.5)	201 (33.6)	12 (21.1)	
Yes	443 (67.5)	398 (66.4)	45 (78.9)	
Neoadjuvant/perioperative therapy regime				0.057
CROSS	232 (52.4)	209 (52.5)	23 (40.4)	
FLOT	78 (17.6)	66 (16.6)	12 (21.2)	
Other	133 (30.0)	123 (30.9)	10 (17.5)	
(y)pT				0.076
1	121 (18.4)	116 (19.4)	5 (8.8)	
2	109 (16.6)	102 (17.0)	7 (12.3)	
3	409 (62.3)	367 (61.3)	42 (73.7)	
4	17 (2.6)	14 (2.3)	3 (5.3)	
(y)pN				**0.016**
0	266 (40.5)	245 (40.9)	21 (36.8)	
1	194 (29.6)	184 (30.7)	10 (17.5)	
2	96 (14.6)	86 (14.4)	10 (17.5)	
3	100 (15.2)	84 (14.0)	16 (28.1)	
L				0.616
0	293 (44.7)	271 (45.2)	22 (38.6)	
1	254 (38.7)	230 (38.4)	24 (42.1)	
2	109 (16.6)	98 (16.4)	11 (19.3)	
V				0.474
0	481 (73.3)	443 (74.0)	38 (66.7)	
1	69 (10.5)	61 (10.2)	8 (14.0)	
2	106 (16.2)	95 (15.9)	11 (19.3)	
Pn				0.158
0	420 (64.0)	390 (65.1)	30 (52.6)	
1	132 (20.1)	116 (19.4)	16 (28.1)	
2	104 (15.9)	93 (15.5)	11 (19.3)	

Bold print marks *p*-values below 0.05

**Table 2:** Patient characteristics of the study cohort, whose proteome was examined, of the total cohort and patients with MDM2-non-amplified or MDM2-amplified tumors. (y)pN: pathological lymph node status (after neoadjuvant therapy), (y)pT: pathological tumor status (after neoadjuvant therapy), CROSS: chemoradiotherapy for oesophageal cancer followed by surgery Study, FLOT: fluorouracil, leucovorin, oxaliplatin, docetaxel, L: lymph vessel infiltration, Pn: perineural infiltration, V: blood vessel infiltration

Characteristic	Total	MDM2		
		non-amplified	amplified	
	*n* (%)	*n* (%)	*n* (%)	*p*-value
No. of patients	72 (100)	37 (100)	35 (100)	
Sex				0.631
Male	58 (80.6)	29 (78.4)	29 (82.9)	
Female	14 (19.4)	8 (21.6)	6 (17.1)	
Age				0.361
< 65	45 (62.5)	25 (67.6)	20 (57.1)	
≥ 65	27 (37.5)	12 (32.4)	15 (42.9)	
Median overall survival (months)	22.5	22.8	22.1	
(minimum-maximum)	(3.8–81.3)	(3.8–76.5)	(3.9–81.3)	
Neoadjuvant/perioperative therapy				0.659
No	16 (22.2)	9 (24.3)	7 (20.0)	
Yes	56 (77.8)	28 (75.7)	28 (80.0)	
Neoadjuvant/perioperative therapy regime				0.564
CROSS	25 (44.6)	12 (42.9)	13 (46.4)	
FLOT	19 (33.9)	8 (28.6)	11 (39.3)	
Other	12 (21.4)	8 (28.6)	4 (14.3)	
(y)pT				0.315
1	10 (13.9)	7 (18.9)	3 (8.6)	
2	8 (11.1)	4 (10.8)	4 (11.4)	
3	52 (72.2)	26 (70.3)	26 (74.5)	
4	2 (2.8)	0 (0.0)	2 (5.7)	
(y)pN				0.651
0	25 (34.7)	14 (37.8)	11 (31.4)	
1	17 (23.6)	10 (27.0)	7 (20.0)	
2	10 (13.9)	5 (13.5)	5 (14.3)	
3	20 (27.8)	8 (21.6)	12 (34.3)	
L				0.858
0	34 (47.2)	17 (45.9)	17 (48.6)	
1	35 (48.6)	18 (48.6)	17 (48.6)	
2	3 (4.2)	2 (5.4)	1 (2.9)	
V				0.316
0	59 (81.9)	32 (86.5)	27 (77.1)	
1	10 (13.9)	3 (8.1)	7 (20.0)	
2	3 (4.2)	2 (5.4)	1 (2.9)	
Pn				0.350
0	44 (61.1)	25 (67.6)	19 (54.3)	
1	25 (34.7)	10 (27.0)	15 (42.9)	
2	3 (4.2)	2 (5.4)	1 (2.9)	

**Fig. 1:**
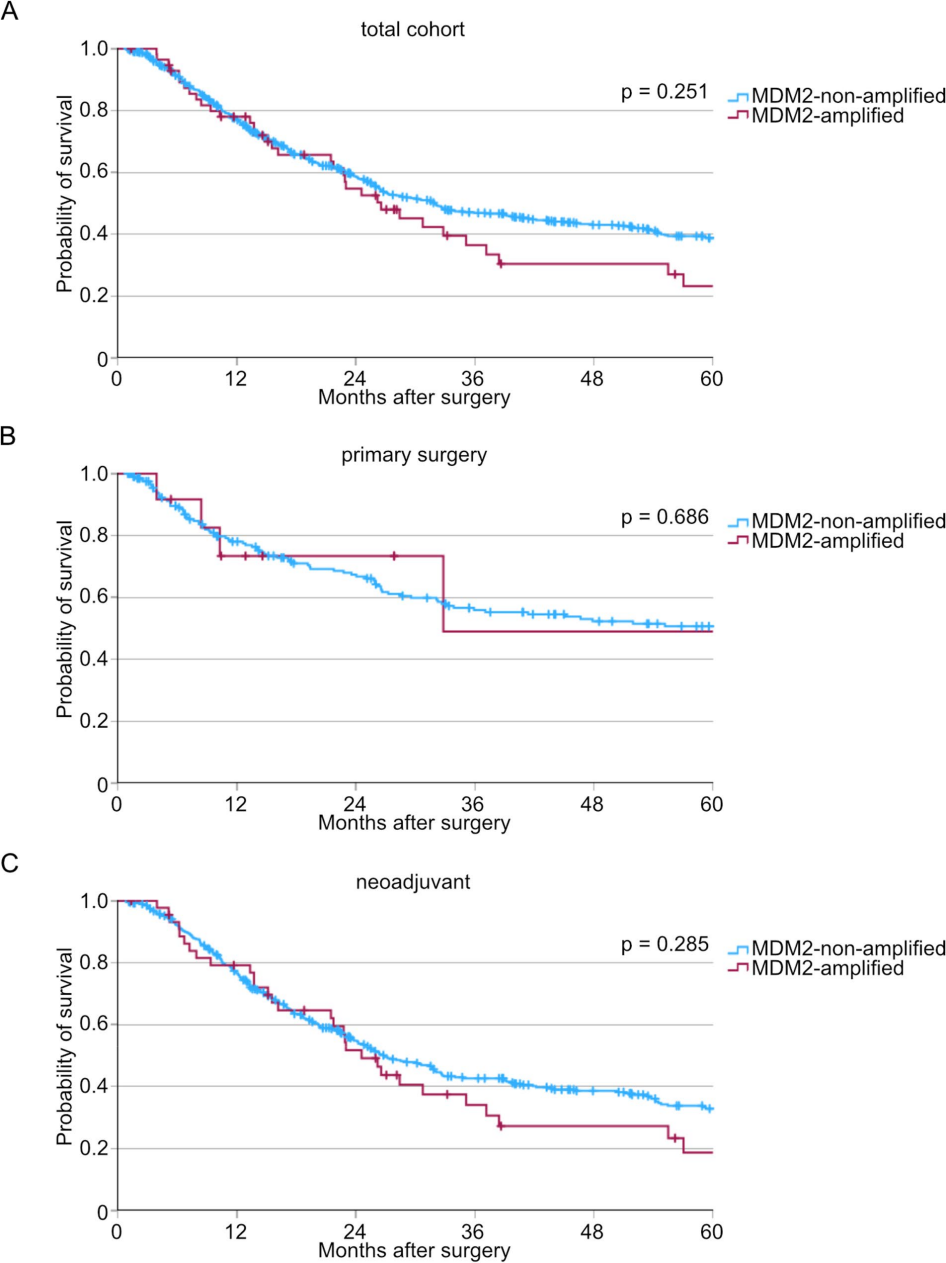
Impact of MDM2 amplification status on patient survival. Kaplan-Meier-curves for (**A**) total cohort (n(MDM2-non-amplified) = 599, n(MDM2-amplified) = 57, *p* = 0.251), (**B**) patients treated with primary surgery (n(MDM2-non-amplified) = 12, n(MDM2-amplified) = 201, *p* = 0.686), and (**C**) patients underwent neoadjuvant treatment (n(MDM2-non-amplified) = 398, n(MDM2-amplified) = 45, *p* = 0.285)

In the total cohort, a significantly higher lymph node status could be observed in MDM2-amplified tumors compared to non-amplified tumors (Table 1). No other significant differences were observed in clinicopathological variables between MDM2-amplified and MDM2-non-amplified patient groups in the total cohort and in our proteome cohort (Tables 1 and 2). Subsequent comparisons of overall survival revealed no significant differences between patients with and without MDM2 amplification (Fig. 1A, *p* = 0.251). As neoadjuvant treatment could have a great impact on survival, we divided the patient cohort into patients who received neoadjuvant or perioperative therapy and a patient cohort who underwent primary surgery. Confirming the previously mentioned results, we could not see a survival difference between patients with or without MDM2 amplification in both subgroups (Fig. 1B + C, p(primary surgery) = 0.686, p(neoadjuvant therapy) = 0.285).

### Various proteins are differentially expressed in MDM2-amplified tumors

**Fig. 2:**
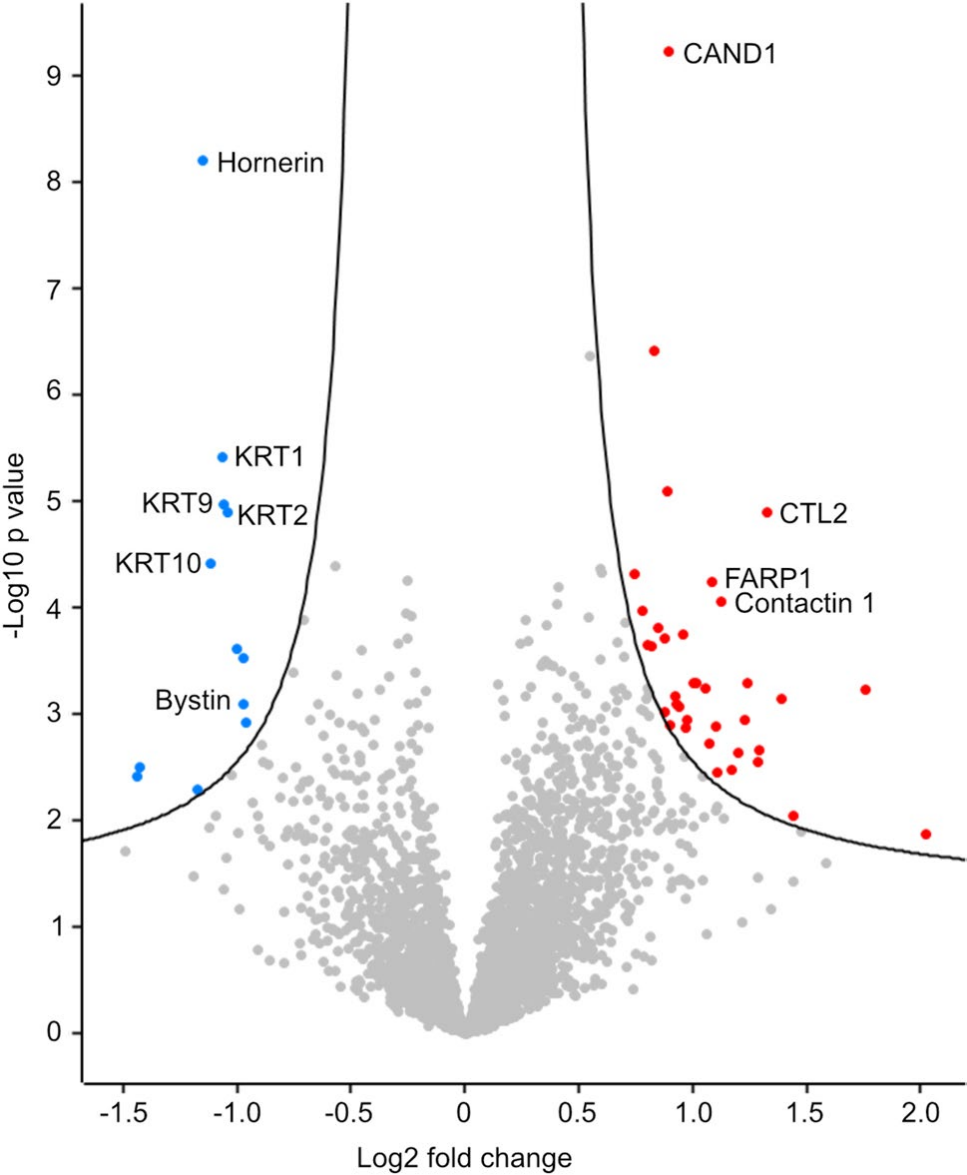
Volcano plot of all detected proteins. Volcano plot of all detected proteins in the total cohort (n(MDM2-amplified) = 35, n(MDM2 non-amplified) = 37). Grey: not significantly different expressed, blue: significantly decreased expression in MDM2-amplified tumors, red: significantly increased expression in MDM2-amplified tumors

To identify differentially expressed proteins in MDM2-amplified tumors compared to MDM2-non-amplified tumors, ANOVA and Welch`s T-test were performed in the total cohort. Here, 48 proteins could be identified that have an increased or decreased protein expression in MDM2-amplified tumors (Fig. 2, Supplementary Table 2). Among others, the expression of Hornerin, Bystin, and several subtypes of keratins (KRT1, KRT2, KRT9, KRT10) were significantly decreased. On the other hand, Choline transporter-like protein 2 (CTL2), Cullin-associated NEDD8-dissociated protein 1 (CAND1), FAD- and RhoGTPase-binding protein 1 (FARP1), and Contactin 1 were significantly upregulated in MDM2 amplified patients.

### MDM2-amplified tumors exhibit a downregulation of immune system-activating pathways

Next, we conducted enrichment analyses to further decipher functional differences between MDM2-amplified and MDM2-non-amplified tumors. Using the three above-mentioned encyclopedias, 37 pathways were found to be downregulated in MDM2-amplified tumors, and 39 pathways were upregulated (*p* < 0.05, Supplementary Table 3). For clarity, we divided pathways into functional groups.

**Fig. 3:**
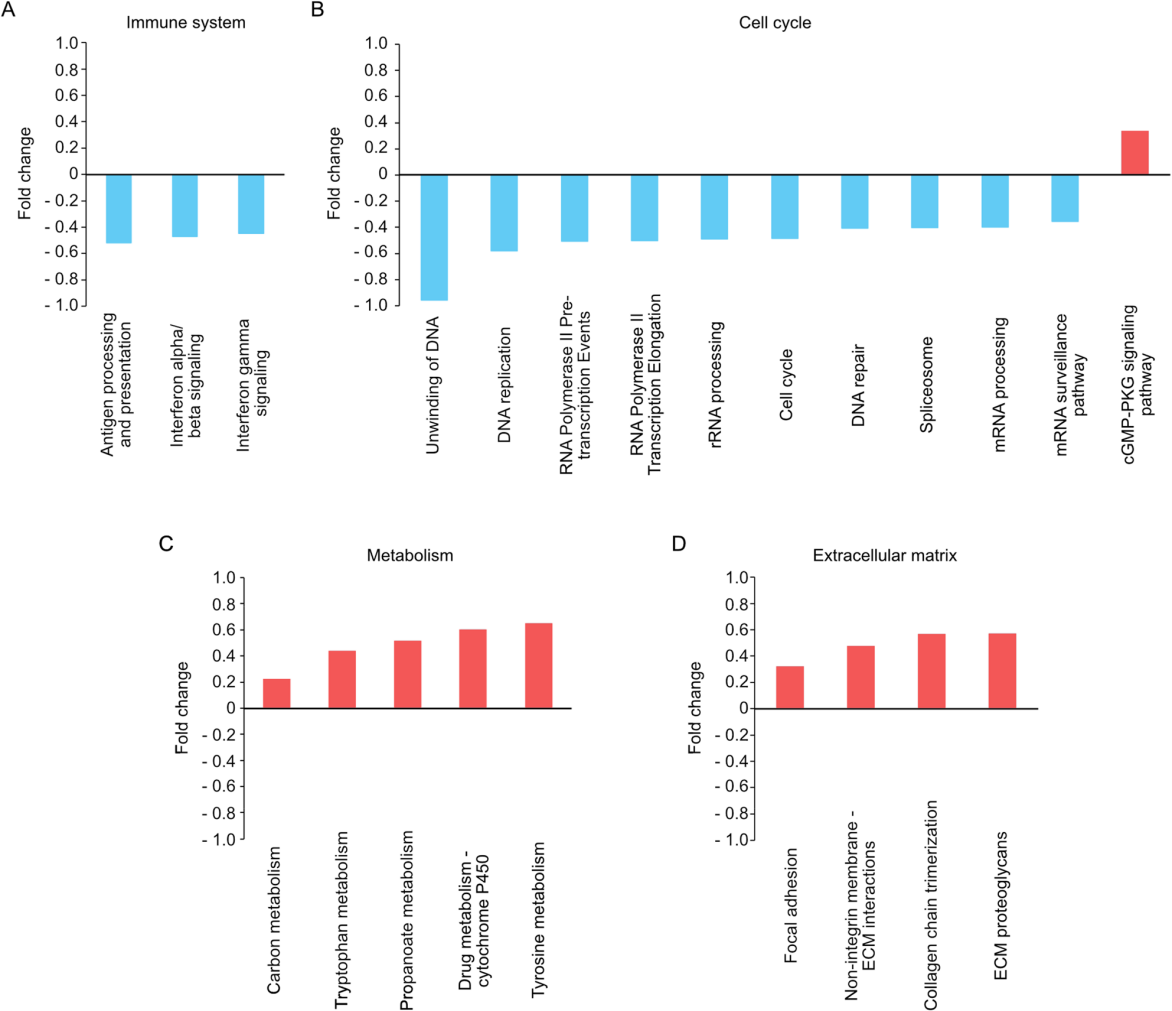
Column charts of differentially expressed pathways. Column charts with differentially expressed pathways. Pathways involved (**A**) in the immune system, (**B**) in the cell cycle, (**C**) in metabolism, and (**D**) in the extracellular matrix. Blue: significantly decreased expression in MDM2-amplified tumors, red: significantly increased expression in MDM2-amplified tumors

First, we analyzed pathways that are involved in regulating the immune system (Fig. 3A). Signaling pathways involved with Interferon alpha, beta, and gamma showed to be downregulated. Furthermore, antigen processing and presentation were also downregulated, leading to a downregulation of the immune system in MDM2-amplified tumors.

### DNA repair pathways are downregulated

Second, we investigated pathways that are involved in the cell cycle, particularly in DNA or RNA processing (Fig. 3B). Here, DNA unwinding and DNA replication pathways were downregulated. On the RNA level, RNA polymerase II pathways, as well as messenger RNA (mRNA) processing and splicing pathways, were inhibited. Decreased ribosomal RNA (rRNA) processing could be detected. Moreover, control and maintenance pathways like DNA repair or mRNA surveillance pathways were underexpressed. Anti-apoptotic pathways like the cyclic guanosine monophosphate-protein kinase G (cGMP-PKG) signaling pathway were upregulated.

### MDM2-amplified tumors show an activated metabolism

Third, we analyzed metabolic pathways (Fig. 3C). Here, carbon metabolism and propanoate metabolism were significantly upregulated. Additionally, amino acid metabolism pathways, including tryptophan and tyrosine metabolism, exhibited increased activity. Notably, xenobiotic metabolism by cytochrome P450 was also enhanced.

### The extracellular matrix differs in MDM2-amplified tumors

Fourth, the composition and the interaction with the extracellular matrix were examined (Fig. 3D). Collagen chain trimerization and extracellular proteoglycans were upregulated. Furthermore, focal adhesions and receptor interactions with the extracellular matrix were enhanced. In further detail, Elastin, Laminin subunit alpha-5, Lysyl oxidase homolog 1, and vascular adhesion protein (VAP-1) were significantly upregulated in MDM2-amplified tumors.

### Subgroup analyses of the proteome

As neoadjuvant therapy could influence the proteome of tumors, we conducted above-mentioned analyses in following subgroups: neoadjuvantly treated MDM2-amplified tumors versus neoadjuvantly treated MDM2-non-amplified tumors, primary resected MDM2-amplified tumors versus primary resected MDM2-non-amplified tumors, and as an internal control, MDM2-amplified tumors versus non-tumoral tissue.

### Differences of neoadjuvant treated MDM2-amplified and non-amplified tumors

Here, we examined the proteome of 28 MDM2-amplified and 28 non-amplified tumors. In these neoadjuvant-treated tumors, we could identify three proteins that were expressed significantly differently (Supplementary Fig. 2, Supplementary Table 4). Hornerin was downregulated. In contrast, CTL2 as well as Aflatoxin B1 aldehyde reductase member 2 (AKR7A2) were upregulated. These proteins have also been identified as significantly different in the analyses of the total cohort. Furthermore, we conducted enrichment analyses (Supplementary Table 5). Here, we could confirm that MDM2-amplified tumors show an altered expression of pathways involved in the cell cycle (Downregulation: Unwinding of DNA, DNA replication, RNA Polymerase II Pre-transcription events, rRNA processing, cell cycle, DNA repair, spliceosome, mRNA processing, mRNA surveillance pathway; upregulation: cGMP-PKG signaling pathway), metabolism (Upregulation: carbon metabolism, tryptophan metabolism, propanoate metabolism, drug metabolism - cytochrome P450, tyrosine metabolism), and the extracellular matrix (Upregulation: focal adhesion, ECM proteoglycans). In contrast, besides the confirmed downregulation of antigen processing and presentation pathways, no significant difference in pathways involved in the interferon alpha, beta, or gamma signaling could be detected.

### Differences of primarily resected MDM2-amplified and non-amplified tumors

Then, we compared the proteome of 7 MDM2-amplified and 9 non-amplified tumors. Here, no significant differences in protein expression could be detected (Supplementary Table 6). Following this, we compared the expression of pathways using enrichment analyses (Supplementary Table 7). We could detect those pathways involved with the immune system (type I interferon signaling pathways, interferon alpha/beta signaling, antigen processing and presentation), and pathways involved with the cell cycle (rRNA processing, mRNA splicing, spliceosome) showed a significant downregulation. On the contrary, the tyrosine metabolism and the PI3K-Akt signaling pathway were significantly upregulated. Moreover, pathways involved with the extracellular matrix (focal adhesion, extracellular matrix organization, extracellular matrix receptor interaction, proteoglycans) were also upregulated.

### Differences of primarily resected MDM2-amplified and non-tumoral tissue

As an internal control, MDM2-amplified tumors, which were resected primarily (*n* = 7), were compared with non-tumoral tissues (*n* = 20). Here, 826 differently expressed proteins could be detected. Six hundred eighty-eight proteins were upregulated, and 138 proteins were downregulated (Supplementary Table 8). Of the above-mentioned upregulated proteins in MDM2-amplified tumors compared to non-amplified tumors, only FARP1 was significantly upregulated in primarily resected MDM2-amplified tumors compared to non-tumoral tissue. On the contrary, neither Hornerin nor Bystin were significantly downregulated compared to non-tumoral tissue. Investigating the enrichment of pathways, 130 differently expressed pathways could be detected (Supplementary Table 9). Here, cell cycle-related pathways like DNA replication, mRNA processing, or mRNA splicing were upregulated. In contrast, metabolic pathways like the citrate cycle, tryptophan metabolism, carbon metabolism, tyrosine metabolism, or pyruvate metabolism were significantly downregulated.

## Discussion

The aim of this study was to investigate the differences between esophageal adenocarcinomas with and without *MDM2* amplification on the proteome level. Therefore, we screened 656 tumor samples for *MDM2* amplification using FISH. Here, 8.7% of the screened tumor samples showed an *MDM2* amplification. This frequency aligns with previously published works [7, 8, 38]. In our patient cohort, no influence of the MDM2 amplification status on patient survival could be detected. This contradicts reports from other cancer types. Exemplary, the occurrence of MDM2 amplification correlated with worse overall survival in non-small lung cancer and esophageal squamous cell cancer [39, 40]. In esophageal adenocarcinoma, a shorter progression-free survival was reported for patients with MDM2-amplified tumors [22]. However, a significantly higher lymph node status could be found in MDM2-amplified tumors in our cohort.

The comparison of the tumor proteomes with and without MDM2 amplification revealed 48 differentially expressed proteins. Here, Hornerin, Bystin, and several keratins (KRT1, KRT2, KRT9, KRT10) showed a significantly decreased expression. Hornerin is part of the S100 protein family and was linked to tumor progression in breast cancer and hepatocellular carcinoma [41, 42]. Moreover, Hornerin mediates angiogenesis in a vascular endothelial growth factor-independent manner (VEGF). This could explain a therapy escape mechanism from anti-angiogenic treatment agents [43]. However, this pathway seems to be downregulated in MDM2-amplified esophageal adenocarcinomas. Bystin is a cytoplasmic protein that builds a complex with Tastin and Trophinin involved in cell adhesion. It is known to play a role in early embryogenesis [44]. In prostate cancer cell lines, Bystin is overexpressed when co-cultured with neurons. This implies a crucial role of Bystin in perineural invasion of prostate cancer cells [45, 46]. However, the roles of both proteins, Bystin and Hornerin, have not been well investigated yet. Further research needs to address the clinical significance of the underexpression of Bystin and Hornerin in MDM2-amplified tumors.

Thirty-six proteins showed an overexpression in MDM2-amplified esophageal adenocarcinomas. Among others, Choline transporter-like protein 2 (CTL2), Cullin-associated NEDD8-dissociated protein 1 (CAND1), FAD- and RhoGTPase-binding protein 1 (FARP1), Aflatoxin B1 aldehyde reductase member 2 (AKR7A2), and Contactin 1 show an elevated expression level. CTL2 is a Na^+^-independent transporter of choline [47]. CTL-mediated high choline levels are linked to increased cell proliferation in various cancer types [48]. CTL2 is overexpressed in esophageal squamous cancer cell lines [49]. Interestingly, CTLs can be inhibited selectively by hemicholinium-3 [48]. Therefore, CTLs are discussed as a possible therapeutic target. CTL1 inhibitors are currently under investigation in cancer cell lines. Here, reduced cell viability and increased caspase 3/7 activity could be observed in tongue squamous cancer cell lines and pancreatic cancer cell lines, respectively. The inhibition of CTL1 results in apoptosis-mediated cell death [50, 51]. While CTL1 inhibitors are already under investigation, the potential inhibition of CTL2 is mainly unknown. Our results encourage the investigation of CTL2 as a possible therapeutic target for patients with MDM2-amplified esophageal adenocarcinoma.

CAND1 is a mediator of Cullin-RING ubiquitin ligases, which is the largest family of E3 ubiquitin ligases. Ubiquitin marks proteins for degradation by proteasomes. It is a positive regulator of the complex’s activity and acts as a degradation promoter [52, 53]. CAND1 overexpression was linked to decreased overall survival in breast and prostate cancer [54, 55]. The knockdown of CAND1 results in decreased cell viability, proliferation, and induced apoptosis in drug-resistant prostate cancer cell lines [55].

FARP1 is a guanine nucleotide exchange factor for RhoGTPases and plays a role in neuronal development as well as in signal regulation and dendritic growth [56]. Pathophysiologically, increased FARP1 levels are linked to reduced patient survival in cutaneous melanoma and gastric cancer [57, 58]. Interestingly, elevated FARP1 expression leads to an upregulation of the mitogen-activated protein kinase (MAPK) pathway [58]. This could be a possible therapeutic target for MDM2-amplified esophageal adenocarcinomas. FDA-approved and clinically used inhibitors of the MAPK pathway exist. A phase III trial with patients with BRAF-mutated melanoma showed a significant prolongation of overall survival if treated with Trametinib, a Mitogen-activated protein kinase kinase (MEK) inhibitor, together with Dabrafenib, a BRAF inhibitor [59]. Further investigations using MDM2-amplified esophageal adenocarcinoma cell lines are needed to evaluate their response to existing MEK inhibitors.

Contactin 1 is a neuronal membrane glycoprotein and is part of the cell adhesion molecules [60]. It is linked to cancer cell proliferation, migration, and invasion in vitro and in vivo in breast cancer [61]. Moreover, its inhibition resulted in reduced cell migration and invasion in gastric cancer [62]. High Contactin 1 expression correlates with poor prognosis in patients with gastric cancer [63]. Therefore, Contactin 1 could also be a potential therapeutic target. In vitro experiments on lung adenocarcinoma cells could show that a downregulation of Contactin 1 led to an increased treatment sensitivity against cisplatin, resulting in increased apoptosis [64]. However, its potential as a therapeutic target requires careful evaluation, as immunoglobulin G4 antibodies against Contactin 1 have been linked to chronic inflammatory demyelinating polyneuropathy [65].

The latter three described proteins are known to correlate with lymph node metastasis in various tumor entities [57, 66, 67]. This could potentially explain, mechanistically, that MDM2-amplified tumors showed significantly more lymph node metastasis compared to non-amplified tumors in our patient cohort. CAND1 was described to impair the type I interferon response, resulting in an immunosuppressive and protumorigenic micromilieu [67]. Furthermore, FARP1 increases cell motility and supports lymph node metastasis [57].

Conducting enrichment analyses, various pathways could be revealed to be up- or downregulated. Highlighting, we observed that MDM2-amplified tumors show an immunosuppressive phenotype. Specifically, interferon signaling as well as antigen processing and presentation are downregulated. The previously mentioned CAND1 is known to support immunosuppression via inhibiting the type I interferon signaling in non-small cell lung cancer [67]. Furthermore, CTL2 was described to show an induced choline uptake after glucocorticoid exposure. This links CTL2 to the mediation of the immunosuppressive effects of glucocorticoids [68]. This suggests that evading immune destruction, one of the hallmarks of cancer, may be a key factor in the pathogenesis of MDM2-amplified esophageal adenocarcinomas [69]. It can be inferred that patients with this type of tumor could additionally benefit from activators of the antitumor immune response. Keeping this in mind, MDM2 amplification is associated with hyperprogression under immunotherapy [21]. Immune resistance is a complex pathomechanism. Inhibition of MDM2 leads to p53-wild-type-dependent changes in the composition of the immune microenvironment. An increase of antigen-presenting dendritic cells and CD8^+^ T cells could be observed in colorectal mouse models. Interestingly, these changes could not be detected in p53-knockout mice. Furthermore, combining the mdm2 inhibitor with a PD-1/PD-L1 blockage could lead to increased complete tumor regression rates. Importantly, this therapy response could not be observed in p53-mutated or knockout tumor models [24]. In our experience, MDM2-amplified esophageal adenocarcinomas are p53-wild type [70]. Indicating that additional MDM2 inhibition could be used as a potential therapeutic target.

We detected a wide range of differentially expressed pathways, including the cell cycle, DNA as well as mRNA pathways, and DNA repair mechanisms. Among these, the significant downregulation of mRNA splicing, rRNA processing, and DNA replication suggests a broader suppression of transcriptional and translational activity in MDM2-amplified tumors. This aligns with the role of MDM2 in repressing p53-mediated transcription, potentially reducing overall protein synthesis and altering tumor cell proliferation dynamics. Further studies are needed to investigate whether this suppression contributes to therapy resistance or other phenotypic characteristics of MDM2-amplified tumors.

As MDM2, the counterpart of p53, is upregulated in MDM2-amplified tumors, this could explain the extensive variation in these pathways [71]. MDM2 overexpression is known to be linked with an anti-apoptotic phenotype [72]. Supporting our results, MDM2 binds to the DNA double-strand break repair complex and inhibits DNA repair, resulting in genomic instability [73, 74].

Moreover, our results showed an upregulation of cytochrome P450 drug metabolism in MDM2-amplified tumors. This is especially interesting since MDM2-amplified tumors exhibit a plurality of therapy resistances [75]. Exemplary, Docetaxel, a part of the recommended chemotherapy regimen for patients with esophageal adenocarcinoma (FLOT), is metabolized by cytochrome P450 enzymes. Since these Cytochrome P450 enzymes are expressed by the tumor cells themselves, this could cause resistance against the antitumoral effect of Docetaxel [1, 76]. This could lead to two potential therapy adjustments after further studies. The recommendation for the administration of Docetaxel could be withdrawn for patients with MDM2-amplified tumors since the tumor cells developed a therapy resistance mechanism against Docetaxel. Second, the additional administration of a cytochrome P450 inhibitor could be recommended to overcome the therapy resistance.

Our data describe an upregulation of distinct metabolic pathways in MDM2-amplified tumors, such as the propanoate, tryptophan, and tyrosine metabolisms. Tryptophan is an essential amino acid involved in various physiological and pathophysiological processes. It is linked to the promotion of neurodegenerative and cancerous diseases [77]. The upregulation of tryptophan metabolism correlates with an increased immune resistance of tumors [78]. The tryptophan metabolism is currently under investigation as a potential therapeutic target. In a phase 1/2 trial, the combination of an indoleamine 2,3-dioxygenase 1 (IDO1) enzyme inhibitor and a programmed cell death protein 1 (PD-1) inhibitor achieved an objective response rate of 55% in patients with various solid tumors [79]. However, no survival benefit could be found in a phase 3 trial in patients with unresectable or metastasized melanoma [80]. This underlines the need for better selection criteria for the administration of tryptophan metabolism inhibitors. We hypothesize that patients with MDM2-amplified esophageal adenocarcinoma would potentially benefit from this treatment. Therefore, further preclinical and clinical studies are needed.

TP53 is by far the most frequently mutated cancer gene in adenocarcinomas and squamous cell carcinomas, with notable regional differences. In a Chinese cancer cohort, 51.4% of analyzed tumors harbored a TP53 mutation, whereas in a cohort from the United States, 35% of cancers showed a TP53 mutation [81, 82]. A Norwegian analysis of 108 specimens of esophageal adenocarcinoma revealed an incidence of TP53 mutation of 28% in esophageal adenocarcinoma [83]. Previous studies reported TP53 mutation rates of 50.5% in esophageal adenocarcinoma [84]. Interestingly, Casson et al. included only treatment-naïve patients, whereas the Norwegian study included 75% neoadjuvant-treated patients [83, 84]. Moreover, TP53 mutations could be observed significantly more frequently in treatment-naïve esophageal adenocarcinomas [83]. This suggests that neoadjuvant therapy may select for TP53-wild-type clones. Since MDM2-amplified EACs are TP53-wild-type, such clones could be enriched in patients who have received neoadjuvant treatment. Notably, 77.8% of the patients in our cohort underwent neoadjuvant therapy prior to analysis. This potential preselection must be taken into account when interpreting our results.

This study also has its limitations. First, our study is solely descriptive. Further preclinical and clinical studies are needed to assess the hypotheses raised. Second, the survival analyses were restricted by their retrospectiveness. Third, we solely investigated the proteome based on the stratification of MDM2-amplification status using FISH. Surely, other elements like mRNAs, phosphorylation status, or the cellular tumor microenvironment influence the tumorigenesis of MDM2-amplified esophageal adenocarcinomas. Further studies are needed to address their differences based on the MDM2-amplification status. Fourth, the subgroup analyses conducted need to be interpreted with caution based on the low patient numbers in the subgroups.

## Conclusions

This study investigated the proteome of MDM2-amplified esophageal adenocarcinomas compared with non-amplified tumors. Interestingly, a plural number of differentially expressed proteins could be described, like an upregulation of FARP1, CTL2, CAND1, and Contactin 1. Here, potential additional treatment options for patients with MDM2-amplified esophageal adenocarcinoma could be described, for example, the inhibition of CTL2 or MEK with Trametinib, which is currently under investigation as an antitumoral treatment agent. Moreover, patients with MDM2-amplified esophageal adenocarcinoma could be eligible for inhibitors of the tryptophan metabolism, like IDO1 enzyme inhibitors. Furthermore, we described that MDM2-amplified tumors are characterized by an immunosuppressive phenotype and an upregulation of drug metabolism via cytochrome P450 enzymes.

We identified several potential therapeutic targets that could serve as additional therapy options, together with MDM2 inhibitors, for patients with MDM2-amplified esophageal adenocarcinoma.

## Supplementary Information

Supplementary Material 1: Supplementary Figure 1. Representative pictures of MDM2 FISH stainings in (A) MDM2 non-amplified tumors, (B) tumors with polysomy without MDM2-amplification, and (C) MDM2-amplified tumors (Zyto-Light SPEC MDM2/CEN12 Dual Core Probe (ZytoVision, Bremerhaven, Germany); green: MDM2, orange: centromere 12; magnification: x63). Scale bar: 10 µm. Supplementary Figure 2. Volcano plot of all detected proteins comparing the proteome of neoadjuvantly treated MDM2-amplified and non-amplified tumors. Grey: not significantly different expressed, blue: significantly decreased expression in neoadjuvantly treated MDM2-amplified tumors, red: significantly increased expression in neoadjuvantly treated MDM2-amplified tumors. Supplementary Table 1. Processed protein expression data from mass spectrometry-based proteomic analysis. This data represents a part of the in PRIDE uploaded raw data set. The rows contain each a unique protein identified by its UniProt identifier. The column names are given the MDM2 amplification status of each sample (MDM2-amplified: MDM2 +; MDM2-non-amplified: NEG.; non tumor tissue: N.G). Additionally, the columns are labeled whether the tumor was treated neoadjuvant (Neoadjuvant) or primarily resected (Primary). These names are not the sample-IDs we used in the PRIDE data set. Supplementary Table 2. This table contains the processed protein expression data and the test results of the ANOVA and Welch`s T-test investigating the whole patient cohort. Each row contains a unique protein. Supplementary Table 3. Test results of the enrichment analyses using the pathway annotation Kyoto Encyclopedia of Genes and Genomes (KEGG) pathway name, Gene Ontology Biological Process (GOBP) name, and Reactome name encyclopedia of the total cohort. Supplementary Table 4. This table contains the processed protein expression data and the test results of the ANOVA and Welch`s T-test investigating the difference of the proteome of neoadjuvantly treated MDM2-amplified versus non-amplified tumors. Each row contains a unique protein. Supplementary Table 5. Test results of the enrichment analyses using the pathway annotation Kyoto Encyclopedia of Genes and Genomes (KEGG) pathway name, Gene Ontology Biological Process (GOBP) name, and Reactome name encyclopedia comparing neoadjuvantly treated MDM2-amplified tumors with non-amplified tumors. Supplementary Table 6. This table contains the processed protein expression data and the test results of the ANOVA and Welch`s T-test investigating the difference of the proteome of primarily resected MDM2-amplified versus non-amplified tumors. Each row contains a unique protein. Supplementary Table 7. Test results of the enrichment analyses using the pathway annotation Kyoto Encyclopedia of Genes and Genomes (KEGG) pathway name, Gene Ontology Biological Process (GOBP) name, and Reactome name encyclopedia comparing primarily resected MDM2-amplified tumors with non-amplified tumors. Supplementary Table 8. This table contains the processed protein expression data and the test results of the ANOVA and Welch`s T-test investigating the difference of the proteome of primarily resected MDM2-amplified versus non-tumoral esophagus tissue. Each row contains a unique protein. Supplementary Table 9. Test results of the enrichment analyses using the pathway annotation Kyoto Encyclopedia of Genes and Genomes (KEGG) pathway name, Gene Ontology Biological Process (GOBP) name, and Reactome name encyclopedia comparing primarily resected MDM2-amplified tumors with non-tumoral esophagus tissue.

## Data Availability

All data supporting the findings of this study are available within the paper, its Supplementary Information, and the PRIDE database.

## Abbreviations

(y)pN: Pathological lymph node status (after neoadjuvant therapy)
(y)pT: Pathological tumor status (after neoadjuvant therapy)
Akt: Protein kinase B
AKR7A2: Aflatoxin B1 aldehyde reductase member 2
CAND1: Cullin-associated NEDD8-dissociated protein 1
CEN: Centromere
cGMP: Cyclic guanosine monophosphate
CROSS: Chemoradiotherapy for Oesophageal Cancer Followed by Surgery Study
CTL: Choline transporter-like protein
DNA: Deoxyribonucleic acid
EAC: Esophageal adenocarcinoma
ECM: Extracellular matrix
et al.: Et alia (and others)
FARP1: FAD- and RhoGTPase-binding protein 1
FISH: Fluorescence in situ hybridization
FLOT: Fluorouracil, Leucovorin, Oxaliplatin, Docetaxel
GOBP: Gene Ontology Biological Process
IDO1: Indoleamine 2,3-dioxygenase 1
KEGG: Kyoto Encyclopedia of Genes and Genomes
KRT: Kreatin
L: Lymph vessel infiltration
MDM: Mouse double minute
MEK: Mitogen-activated protein kinase kinase
mRNA: Messenger ribonucleic acid
PD-1: Programmed cell death protein 1
PKG: Protein kinase G
Pn: Perineural infiltration
RNA: Ribonucleic acid
rRNA: Ribosomal ribonucleic acid
V: Blood vessel infiltration
VAP-1: Vascular adhesion protein
VEGF: Vascular endothelial growth factor
