# Supplementary figures and images for: MDM2-amplified esophageal adenocarcinomas exhibit an activated metabolic and immunosuppressive phenotype with multiple potential therapeutic targets
Source: BMC Cancer. 2025 Nov 26;25:1863. doi: 10.1186/s12885-025-15367-3 (PMC12690831; doi:10.1186/s12885-025-15367-3)

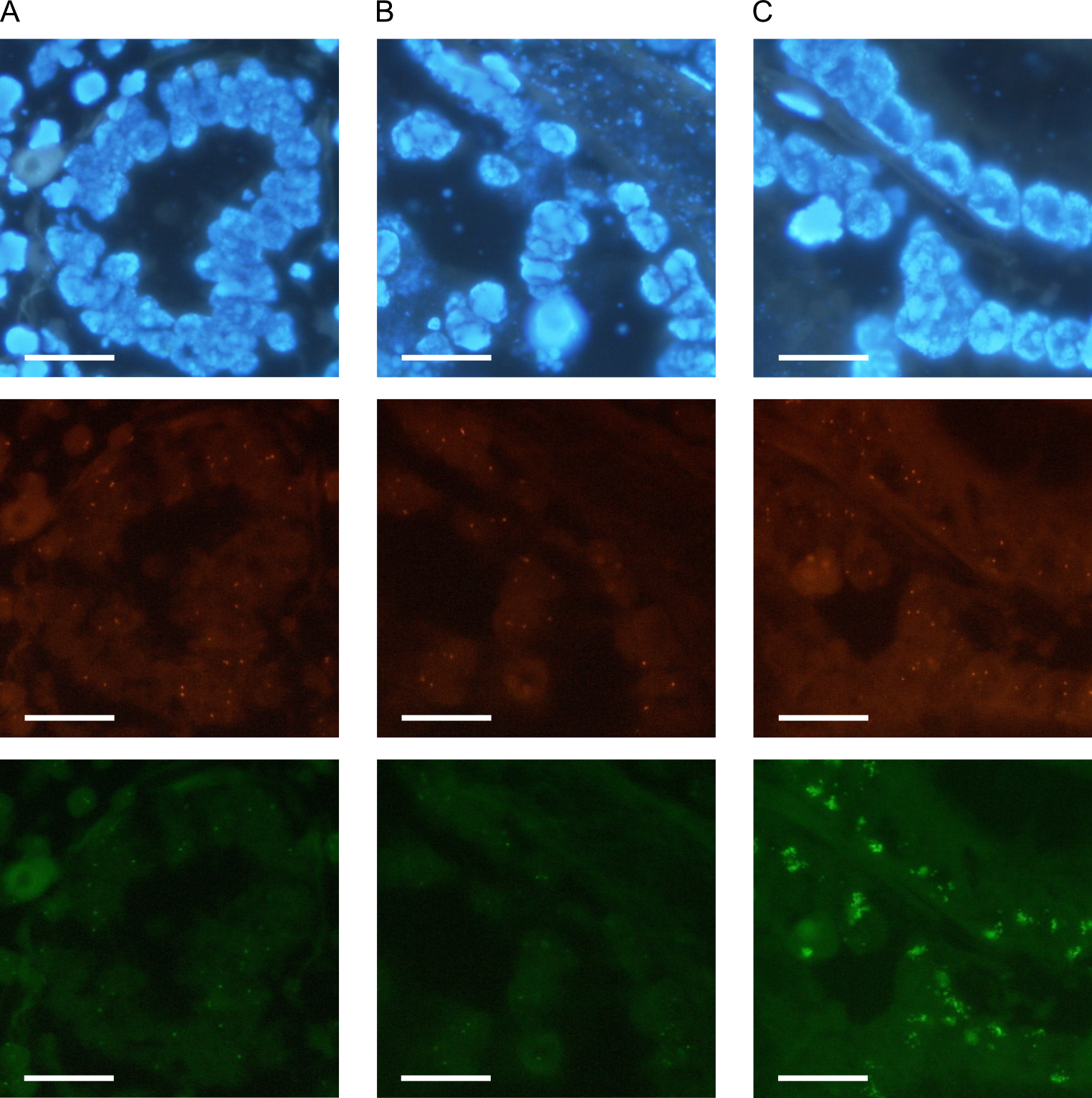

Supplement: Supplementary file 1 — Supplementary Material 1: Supplementary Figure 1. Representative pictures of MDM2 FISH stainings in (A) MDM2 non-amplified tumors, (B) tumors with polysomy without MDM2-amplification, and (C) MDM2-amplified tumors (Zyto-Light SPEC MDM2/CEN12 Dual Core Probe (ZytoVision, Bremerhaven, Germany); green: MDM2, orange: centromere 12; magnification: x63). Scale bar: 10 µm. Supplementary Figure 2. Volcano plot of all detected proteins comparing the proteome of neoadjuvantly treated MDM2-amplified and non-amplified tumors. Grey: not significantly different expressed, blue: significantly decreased expression in neoadjuvantly treated MDM2-amplified tumors, red: significantly increased expression in neoadjuvantly treated MDM2-amplified tumors. Supplementary Table 1. Processed protein expression data from mass spectrometry-based proteomic analysis. This data represents a part of the in PRIDE uploaded raw data set. The rows contain each a unique protein identified by its UniProt identifier. The column names are given the MDM2 amplification status of each sample (MDM2-amplified: MDM2 +; MDM2-non-amplified: NEG.; non tumor tissue: N.G). Additionally, the columns are labeled whether the tumor was treated neoadjuvant (Neoadjuvant) or primarily resected (Primary). These names are not the sample-IDs we used in the PRIDE data set. Supplementary Table 2. This table contains the processed protein expression data and the test results of the ANOVA and Welch`s T-test investigating the whole patient cohort. Each row contains a unique protein. Supplementary Table 3. Test results of the enrichment analyses using the pathway annotation Kyoto Encyclopedia of Genes and Genomes (KEGG) pathway name, Gene Ontology Biological Process (GOBP) name, and Reactome name encyclopedia of the total cohort. Supplementary Table 4. This table contains the processed protein expression data and the test results of the ANOVA and Welch`s T-test investigating the difference of the proteome of neoadjuvantly treated MDM [file 12885_2025_15367_MOESM1_ESM.zip › Suppl_Methods_updated/Supp_Fig_1.tiff]

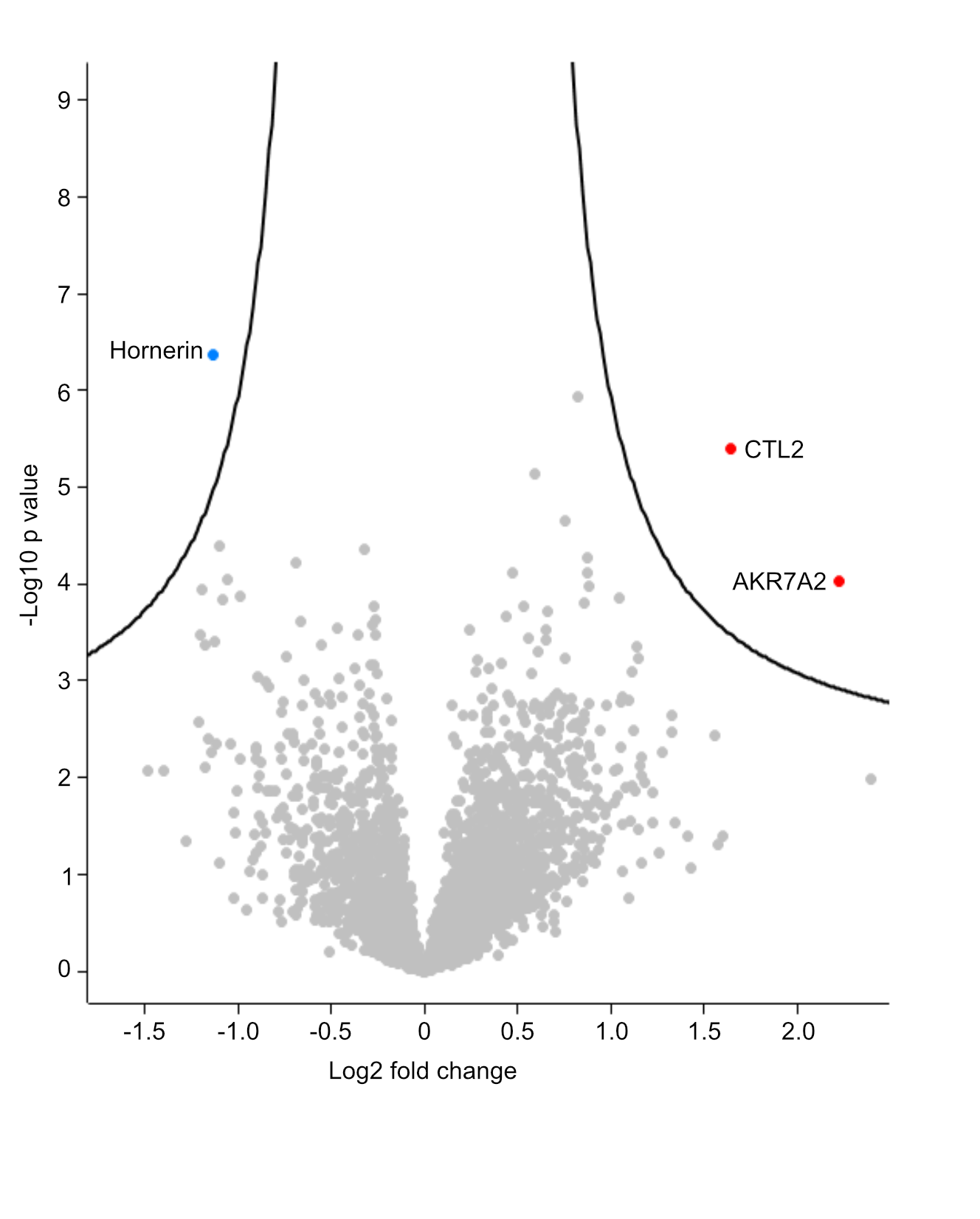

Supplement: Supplementary file 1 — Supplementary Material 1: Supplementary Figure 1. Representative pictures of MDM2 FISH stainings in (A) MDM2 non-amplified tumors, (B) tumors with polysomy without MDM2-amplification, and (C) MDM2-amplified tumors (Zyto-Light SPEC MDM2/CEN12 Dual Core Probe (ZytoVision, Bremerhaven, Germany); green: MDM2, orange: centromere 12; magnification: x63). Scale bar: 10 µm. Supplementary Figure 2. Volcano plot of all detected proteins comparing the proteome of neoadjuvantly treated MDM2-amplified and non-amplified tumors. Grey: not significantly different expressed, blue: significantly decreased expression in neoadjuvantly treated MDM2-amplified tumors, red: significantly increased expression in neoadjuvantly treated MDM2-amplified tumors. Supplementary Table 1. Processed protein expression data from mass spectrometry-based proteomic analysis. This data represents a part of the in PRIDE uploaded raw data set. The rows contain each a unique protein identified by its UniProt identifier. The column names are given the MDM2 amplification status of each sample (MDM2-amplified: MDM2 +; MDM2-non-amplified: NEG.; non tumor tissue: N.G). Additionally, the columns are labeled whether the tumor was treated neoadjuvant (Neoadjuvant) or primarily resected (Primary). These names are not the sample-IDs we used in the PRIDE data set. Supplementary Table 2. This table contains the processed protein expression data and the test results of the ANOVA and Welch`s T-test investigating the whole patient cohort. Each row contains a unique protein. Supplementary Table 3. Test results of the enrichment analyses using the pathway annotation Kyoto Encyclopedia of Genes and Genomes (KEGG) pathway name, Gene Ontology Biological Process (GOBP) name, and Reactome name encyclopedia of the total cohort. Supplementary Table 4. This table contains the processed protein expression data and the test results of the ANOVA and Welch`s T-test investigating the difference of the proteome of neoadjuvantly treated MDM [file 12885_2025_15367_MOESM1_ESM.zip › Suppl_Methods_updated/Supp_ Fig_2.tiff]
